# An A.I. classifier derived from 4D radiomics of dynamic contrast-enhanced breast MRI data: potential to avoid unnecessary breast biopsies

**DOI:** 10.1007/s00330-021-07787-z

**Published:** 2021-03-20

**Authors:** Nina Pötsch, Matthias Dietzel, Panagiotis Kapetas, Paola Clauser, Katja Pinker, Stephan Ellmann, Michael Uder, Thomas Helbich, Pascal A. T. Baltzer

**Affiliations:** 1 Department of Biomedical Imaging and Image Guided Therapy, Medical University of Vienna, Waehringerguertel 18-20, 1090 Vienna, Austria; 2 Institute of Radiology, Erlangen University Hospital, Maximiliansplatz 2, 91054 Erlangen, Germany; 3 Department of Radiology, Breast Imaging Service, Memorial Sloan Kettering Cancer Center, 1275 York Avenue, New York, NY 10065 USA

**Keywords:** Neural network, Principal component analysis, Breast biopsies, Breast MRI, Breast cancer

## Abstract

**Objectives:**

Due to its high sensitivity, DCE MRI of the breast (bMRI) is increasingly used for both screening and assessment purposes. The high number of detected lesions poses a significant logistic challenge in clinical practice. The aim was to evaluate a temporally and spatially resolved (4D) radiomics approach to distinguish benign from malignant enhancing breast lesions and thereby avoid unnecessary biopsies.

**Methods:**

This retrospective study included consecutive patients with MRI-suspicious findings (BI-RADS 4/5). Two blinded readers analyzed DCE images using a commercially available software, automatically extracting BI-RADS curve types and pharmacokinetic enhancement features. After principal component analysis (PCA), a neural network–derived A.I. classifier to discriminate benign from malignant lesions was constructed and tested using a random split simple approach. The rate of avoidable biopsies was evaluated at exploratory cutoffs (C_1_, 100%, and C_2_, ≥ 95% sensitivity).

**Results:**

Four hundred seventy (295 malignant) lesions in 329 female patients (mean age 55.1 years, range 18–85 years) were examined. Eighty-six DCE features were extracted based on automated volumetric lesion analysis. Five independent component features were extracted using PCA. The A.I. classifier achieved a significant (*p* < .001) accuracy to distinguish benign from malignant lesion within the test sample (AUC: 83.5%; 95% CI: 76.8–89.0%). Applying identified cutoffs on testing data not included in training dataset showed the potential to lower the number of unnecessary biopsies of benign lesions by 14.5% (C_1_) and 36.2% (C_2_).

**Conclusion:**

The investigated automated 4D radiomics approach resulted in an accurate A.I. classifier able to distinguish between benign and malignant lesions. Its application could have avoided unnecessary biopsies.

**Key Points:**

*• Principal component analysis of the extracted volumetric and temporally resolved (4D) DCE markers favored pharmacokinetic modeling derived features.*

*• An A.I. classifier based on 86 extracted DCE features achieved a good to excellent diagnostic performance as measured by the area under the ROC curve with 80.6% (training dataset) and 83.5% (testing dataset).*

*• Testing the resulting A.I. classifier showed the potential to lower the number of unnecessary biopsies of benign breast lesions by up to 36.2%, p < .001 at the cost of up to 4.5% (n = 4) false negative low-risk cancers.*

**Supplementary Information:**

The online version contains supplementary material available at 10.1007/s00330-021-07787-z.

## Introduction

Due to its superior sensitivity, dynamic contrast-enhanced (DCE) magnetic resonance imaging (MRI) of the breast (bMRI) is an established diagnostic tool for screening in high-risk patients and problem-solving in equivocal and unclear breast lesions detected by mammography or ultrasound as well as monitoring of response to treatment [1, 2]. Recently, convincing evidence has been published supporting the use of bMRI in intermediate-risk screening such as in women with extremely dense breasts, likely to increase the demand for bMRI examinations in the future [3–5]. In bMRI, the main criterion for identifying suspicious lesions is contrast enhancement. While a lack of contrast enhancement practically excludes cancer, contrast enhancing lesions potentially raise suspicion for malignancy.

The diagnostic challenge in bMRI remains to distinguish between benign and malignant enhancement [6, 7]. In women referred to biopsy due to BI-RADS 4 or 5 findings, a majority of these lesions of 40.2–84.6% yield benign results [8–10]. These false positive findings requiring additional image-guided interventions should be kept to a minimum due to high and expensive demands regarding personnel and magnet time [2]. Therefore, methods for avoiding false positive MR BI-RADS category assignments are warranted. Previous research efforts used either further MRI techniques [11–13] or dedicated clinical decision rules based on morphologic and kinetic BI-RADS criteria [14]. While the results of these approaches were encouraging, additional measurements increase magnet time and clinical decision rules require human feature interpretation. Even though clinical decision rules may reduce image interpretation differences due to different experience levels [15], inter-reader variation remains [16]. To take advantage of the high sensitivity of bMRI without causing too many recalls including biopsy recommendations, computational information–centered A.I. methods such as radiomics and machine learning are desirable. Radiomics is an increasingly important field in medicine, providing imaging-derived markers automatically extracted from large amounts of data that are beyond human recognition [17].

Initial approaches focused on automatized signal-intensity time curve evaluation, demonstrating comparable results as human readers [18]. Williams et al [19] found that semiautomatic software analysis of lesion enhancement kinetics facilitated the interpretation of bMRI exams, leading to a better discrimination of benign and malignant lesions. By applying their software to biopsied lesions, they were able to demonstrate a reduction of the false positive rate (corresponding to avoidable biopsies) by up to 23% using semiautomatic determination of enhancement kinetics. In a methodologically comparable setting, Gweon et al [20] reported a potential reduction of biopsies of benign lesions by 53%. Applied to non-mass lesions, Vag et al [21] also found computer-aided analysis of contrast enhancement kinetics could improve breast cancer diagnosis, though not accurate enough to rely on BI-RADS enhancement kinetics as a single diagnostic criterion. The latter results are in line with multiple publications that support the combination of information from multiple image-derived contrasts and criteria to ensure sufficient diagnostic certainty to support clinical decision-making [6, 10, 22–25].

Notably, as DCE is the backbone of bMRI, hypothesis-driven research has led to well-established pharmacokinetic models, most importantly the Tofts model providing parameters reflecting tissue vascularization properties. One of those parameters *k*_trans_ (i.e., transfer constant of contrast medium (CM) from plasma compartment into the extravascular extracellular space (EES)) reflects the contrast medium influx in the investigated tissue. Malignant lesions show a higher net capillary diameter and a higher vascular permeability leading to higher k_trans_ values as compared to benign lesions. The second parameter is *v*_e_ (i.e., EES per tissue volume) which describes the extracellular extravascular distribution volume. Due to an increased cellularity and desmoplastic changes, it is decreased in malignant lesions. The combination of these two parameters shapes the dynamic enhancement curve and both have been linked to the biological behavior of such characterized tissue [26–28]. Radiomics can combine this physiological information derived from temporally and spatially resolved (= 4D) DCE data with machine learning.

Our objective was to evaluate such a 4D radiomics approach using DCE-bMRI. The diagnostic task was to distinguish benign from malignant enhancing breast lesions for aiding radiologists in clinical decision-making with the aim to avoid unnecessary biopsies.

## Materials and methods

### Study design

This retrospective, single-center, cross-sectional observational diagnostic study was approved by the local ethical review board (Friedrich Schiller Universität Jena), waiving the need for informed consent. The patient***-***related data were de-identified and handled in accordance with standards of good scientific practice. Study design, manuscript editing and reporting of findings, was done with respect to the CLAIM guidelines [29]*.* Data generated or analyzed during the study are available from the corresponding author by request.

### Patients

We included consecutive women who underwent bMRI from 03/2005 to 10/2006 at the department of Institute of Diagnostic and Interventional Radiology, University Hospital Jena, Germany, for suspicious or unclear findings (BI-RADS 0, 4, or 5) in mammography or ultrasound. Mammography and/or ultrasound were either performed for screening reasons or as diagnostic workup in symptomatic women (e.g., palpable lump), hence representing the routinely imaged patient population for staging and problem-solving bMRI [10, 12, 22]. Final multimodal assessment of the included lesions was rated BI-RADS 4 or 5 in a double reading approach of two out of four radiologists with 5–25 years of breast imaging experience. Consequently, all underwent histological verification after bMRI by means of ultrasound-guided 14G core biopsy or MRI-guided 9G console-based vacuum-assisted breast biopsy. All malignant lesions and all lesions of uncertain malignant potential (B3 [30]) underwent surgery. Surgery was also performed in single cases where radio-pathological congruence could not be established (highly suspicious findings with histological results suggesting a missed biopsy target). For the reference standard, histopathological diagnoses were dichotomized into benign vs malignant. Examinations performed after neoadjuvant chemotherapy were excluded from further analysis avoiding bias due to altered enhancement data. The final study dataset contained 329 women with 470 histologically verified lesions.

Patients analyzed for this study have been investigated in previous investigations with different purpose, analyses, and results [18].

### MRI scanner and imaging technique

Imaging was performed according to international standards [1, 31, 32] on clinical 1.5T magnetic resonance imaging units (Magnetom Sonata and Magnetom Symphony, Siemens Healthineers) using dedicated bilateral receive-only 4-channel breast coils. The imaging protocol included 8 dynamic axial T1-weighted spoiled gradient echo (repetition time 113 ms, echo time 5 ms, flip angle 80°, spatial resolution 1.1 × 0.9 × 3 mm, 33 slices, interslice gap depending on breast size 0–20%, temporal resolution 60 s) measurements, one before and 7 after IV contrast media (0.1 mmol/kg of Gd-DTPA). The contrast medium was administered intravenously as a rapid bolus (3 mL/s), by an automatic injector (Spectris, Medrad). Subtractions of precontrast images from the postcontrast dynamic images were performed automatically by the scanner software.

### Image analysis

All image data was analyzed by commercially available software (currently available as DynaCAD, a class 2 FDA cleared medical product, registration number 892.2050). Data analysis was performed by two readers blinded towards the histopathological outcome supervised by a breast imaging expert (P.B.). Readers received special training (*n* = 300 independent exams with histological verification) both in bMRI and in handling the software.

### Preprocessing and lesion segmentation

After transfer of the non-manipulated DICOM data via the local Picture Archiving and Communication System (PACS), preprocessing included automated elastic motion registration. The registered dynamic series were color-coded using thresholds for initial and delayed phase enhancement using one pre- (P^0^) and two postcontrast time points (P^1^ early, P^2^ delayed after 1 min and 7 min, respectively). The initial change in signal intensity (wash-in) from P^0^ to P^1^ was required to pass a threshold of 33% relative signal increase. If this threshold was passed, the early phase enhancement could be categorized as follows: (i) 33–50% (slow), (ii) > 50–100% (medium), and (iii) > 100% (fast) signal increase. The curve type was further categorized by the delayed enhancement between P^1^ and P^2^ as follows: (i) persistent increase (> 10% signal increase), (ii) plateau (stable signal ± 10%), and (iii) wash-out (> 10% signal decrease). These criteria gave a total of 9 curve type combinations (see supplemental digital content 1 for illustration of curve types). Voxels not passing the initial enhancement threshold were excluded from the analysis. Pharmacokinetic mapping was performed using the Tofts model with population-based arterial input function and T1 time.

**Fig. 1:**
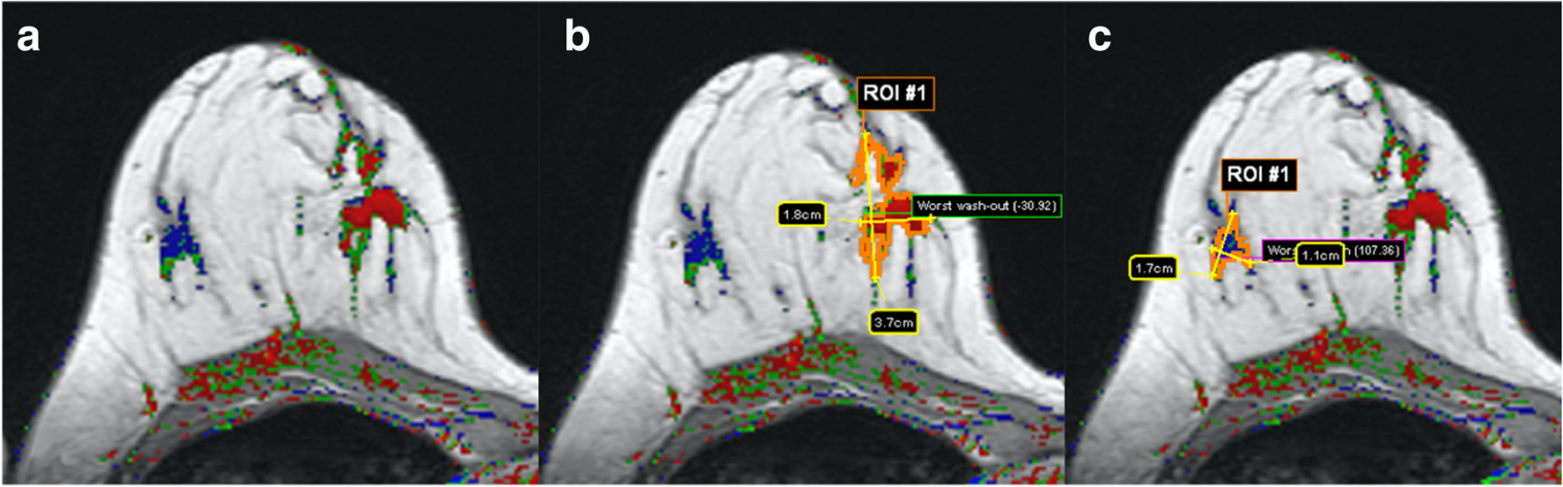
Example for automated lesion segmentation in a 49-year-old woman with her2 type invasive breast cancer not otherwise specified (NST) in the medial right breast (**a**, coded red on the parametric map). After marking the lesion by a single mouse-click, an irregular mass is accurately delineated in a volumetric manner (**b**, only one slice shown here). Subsequently, the ultimately benign lesion (**c**, lateral right breast) is segmented automatically after marking it with one mouse-click

Enhancing lesions were segmented in a supervised manner using an automated multislice 3D segmentation procedure provided by the software (Fig. 1). The interaction with the software was by manually selecting a lesion for analysis by clicking on it. If the automated segmentation failed in single cases due to diffuse, extensive enhancements, a manual segmentation could be performed. Segmentation results were controlled by the study supervisor based on multimodal imaging data and histopathological reports (P.B.).

### Image data extraction

After lesion segmentation, the software displayed the following image features, yielding a total of 86 parameters, which were used for further evaluation and diagnostic model building.

1. Pre-contrast T1w signal intensities and signal intensities of all threshold-passing voxels at all time points after CM injection (*n* = 8, mean curve)
2. Automatically chosen voxel clusters (3 by 3) within the whole segmented lesion presenting the most suspicious curve types:
  - Maximum wash-in curve signal intensities (including one precontrast scan, *n* = 8)
  - Relative maximum wash-out curve signal intensities (*n* = 7)
  - Relative maximum wash-in/wash-out curve signal intensities (*n* = 7)
3. Distribution of subvolume percentages defined by curve types 1–9 (*n* = 9; e.g., percentage of medium wash-out voxels within the lesion)
4. Voxel-wise distribution (percentiles 10 to 90 and quartiles) of pharmacokinetic parameters derived from the Tofts model (*n* = 33, iAUC, *k*_trans_, *v*_e_)

Consequently, results were exported into a database and additional secondary parameters were calculated (Excel in Office 365, Microsoft, US):

5. Relative wash-out rates (defined as: _rel_SI^initial^–_rel_SI^delayed^) using the first and second (peak) postcontrast time points as reference points, leading to two values per curve (*n* = 8; mean, maximum wash-in, maximum wash-out, maximum wash-in/wash-out)
6. Overall lesion percentage of wash-out (i–iii/III), plateau (i–iii/II) and persistent (i–iii/I) curve types (*n* = 3).
7. Interquartile ranges for iAUC, k_trans_ and v_e_ (*n* = 3).

**Fig. 2:**
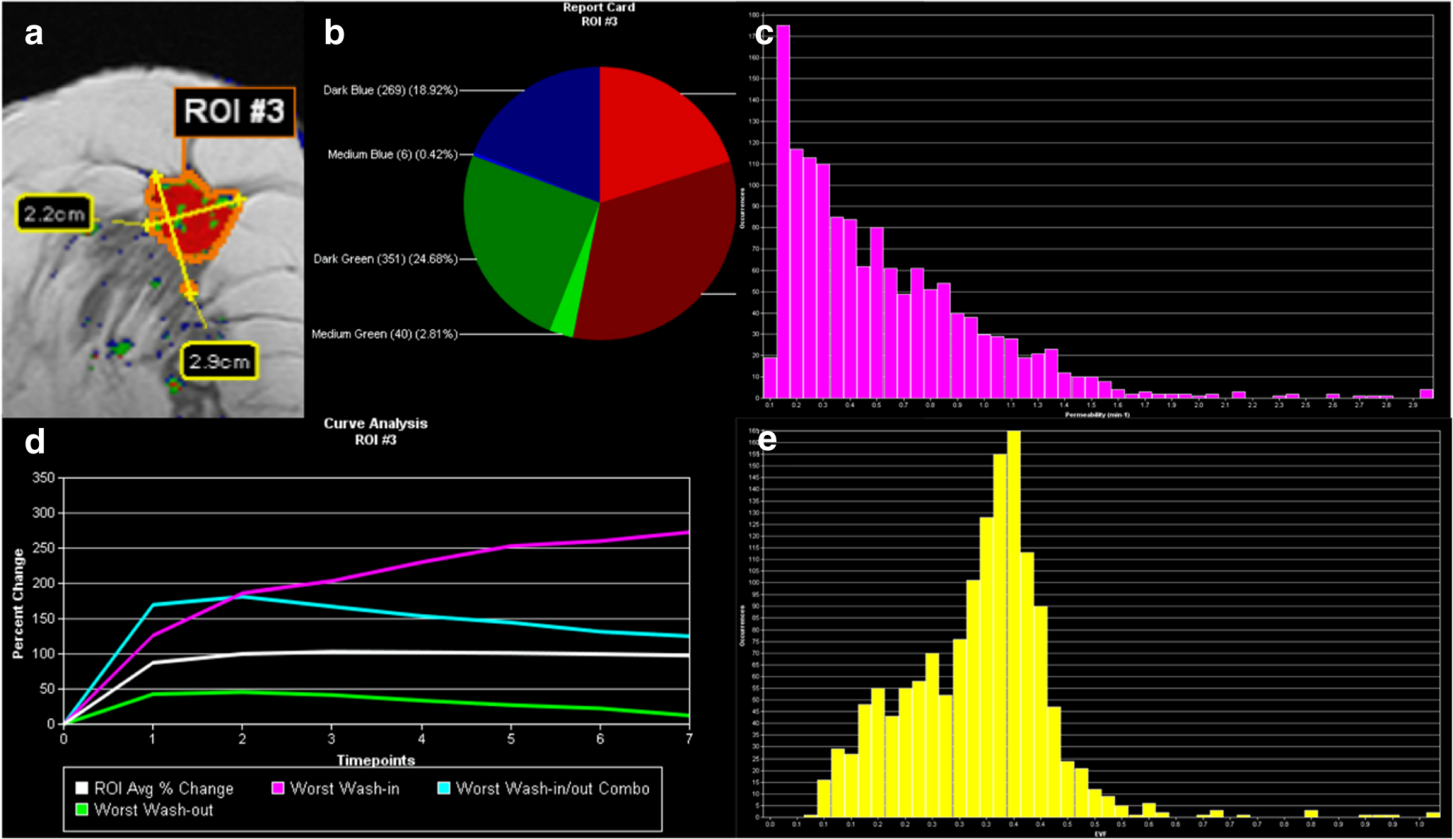
Visualization example of the volumetric analysis of a poorly differentiated (high grade, G3) invasive ductal cancer, not otherwise specified (NST) in a 54-year-old woman. **a** The segmentation also shown in Fig. 1, (**b**) the distribution of enhancement curve types as defined in the methods section (red: wash-out; green: plateau enhancement; blue: persistent enhancement; the shades denote the initial enhancement: dark: slow, intermediate: medium, bright: fast). **c** A histogram of *k*_trans_ while E shows a histogram of *v*_e_ values. The signal-intensity time curves for the whole lesion (white), the maximum initial enhancement (purple), the maximum wash-out (green), and the maximum initial enhancement to wash-out curve (turquoise) are shown in **d**. The figure presents some of the visualization methods provided by the software used for image data analysis. All raw data were exported voxel-wise for further analysis as specified in the methods section. The A.I. classifier provided a pseudo-probability of malignancy of 77% which was above both C_1_ and C_2_ thresholds

**Fig. 3:**
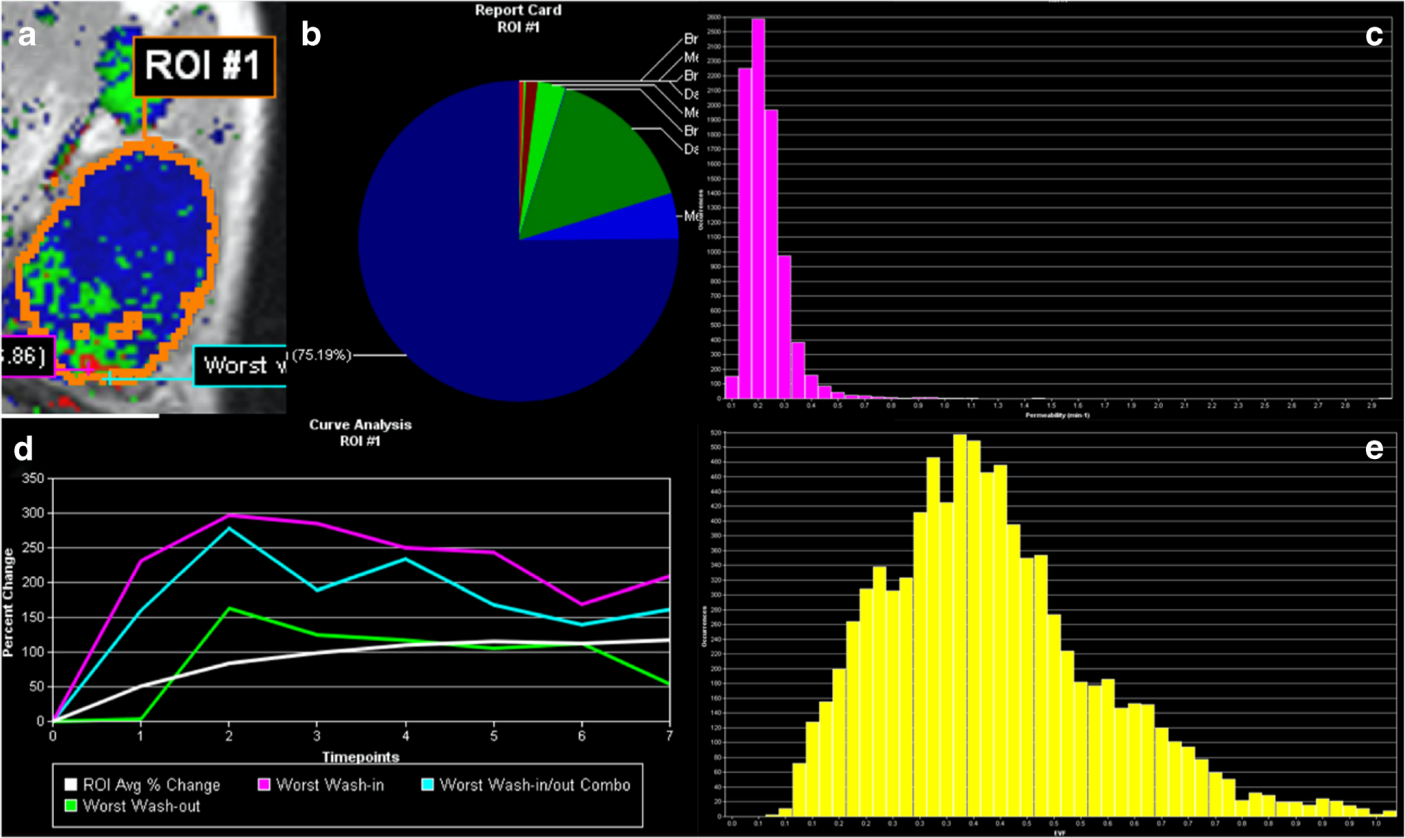
Visualization example of the volumetric analysis of a fibroadenoma B2 (benign finding in biopsy, no further procedure needed) in a 34-year-old woman. **a** The segmentation also shown in Fig. 1, (**b**) the distribution of enhancement curve types as defined in the methods section (red: wash-out; green: plateau enhancement; blue: persistent enhancement; the shades denote the initial enhancement: dark: slow, intermediate: medium, bright: fast). **c** A histogram of *k*_trans_, **e** a histogram of *v*_e_ values. The signal-intensity time curves for the whole lesion (white), the maximum initial enhancement (purple), the maximum wash-out (green), and the most suspect curve (turquoise) are shown in **d**. The figure presents some of the visualization methods provided by the software used for image data analysis. All raw data were exported voxel-wise for further analysis as specified in the methods section. The A.I. classifier provided a pseudo-probability of malignancy of 6% which was below both C_1_ and C_2_ thresholds

Examples for malignant and benign lesions are given in Fig. 2 and Fig. 3.

### Data dimension reduction and diagnostic model building

Principal component analysis using all 86 extracted parameters was used for data dimension reduction. An eigenvalue cutoff of 3 as suggested by our statistician was set and all components showing higher eigenvalues saved for further analysis and model building. To build a diagnostic A.I. classifier, an artificial neural network (ANN) using multilayer perceptron architecture was trained. The input layer consisted of the principal component analysis (PCA) extracted components, the output layer was the probability of malignancy in a binary benign vs malignant task. The ANN architecture including the number and nodes of hidden layers, activation function (hyperbolic tangent or sigmoid), and the number of training epochs was automatically chosen based on classification performance improvement. The initial constraints for the number of units within the hidden layer was set to range between one and 50. Training was done in batch mode using the scaled conjugant grading algorithm for optimization. Initial lambda was set to 5 × 10^−7^, initial sigma to 5 × 10^−5^. The number of training epochs was automatically chosen with the minimum relative change in training error set to 0.0001 and the minimum relative change in training error ratio set to 0.001. The A.I. classifier was trained on 70% of the cases, leaving 30% as an independent testing sample out of the same data source. All calculations were performed using SPSS version 25, 2017 (SPSS Inc., IBM).

### Diagnostic performance statistics

The diagnostic performance of the constructed A.I. classifier to distinguish benign from malignant breast lesions as determined by histopathology as the reference standard was assessed using ROC analysis. The difference of the calculated AUCs against chance was tested and considered significant if *p* ≤. 05. Cutoffs with high sensitivity (100%, C_1_; ≥ 95%, C_2_) were identified in the training dataset and then applied on the testing dataset to estimate the potential of the A.I. classifier to avoid unnecessary biopsies which equals the specificity because the patient population consisted only of suspicious biopsied findings. At the same time, the number of missed (false negative) cancers at these cutoffs could be determined. Medcalc version 19, 2019 (Medcalc Software Ltd.) was used for all ROC analyses.

## Results

### Dataset: patients and lesions

**Fig. 4:**
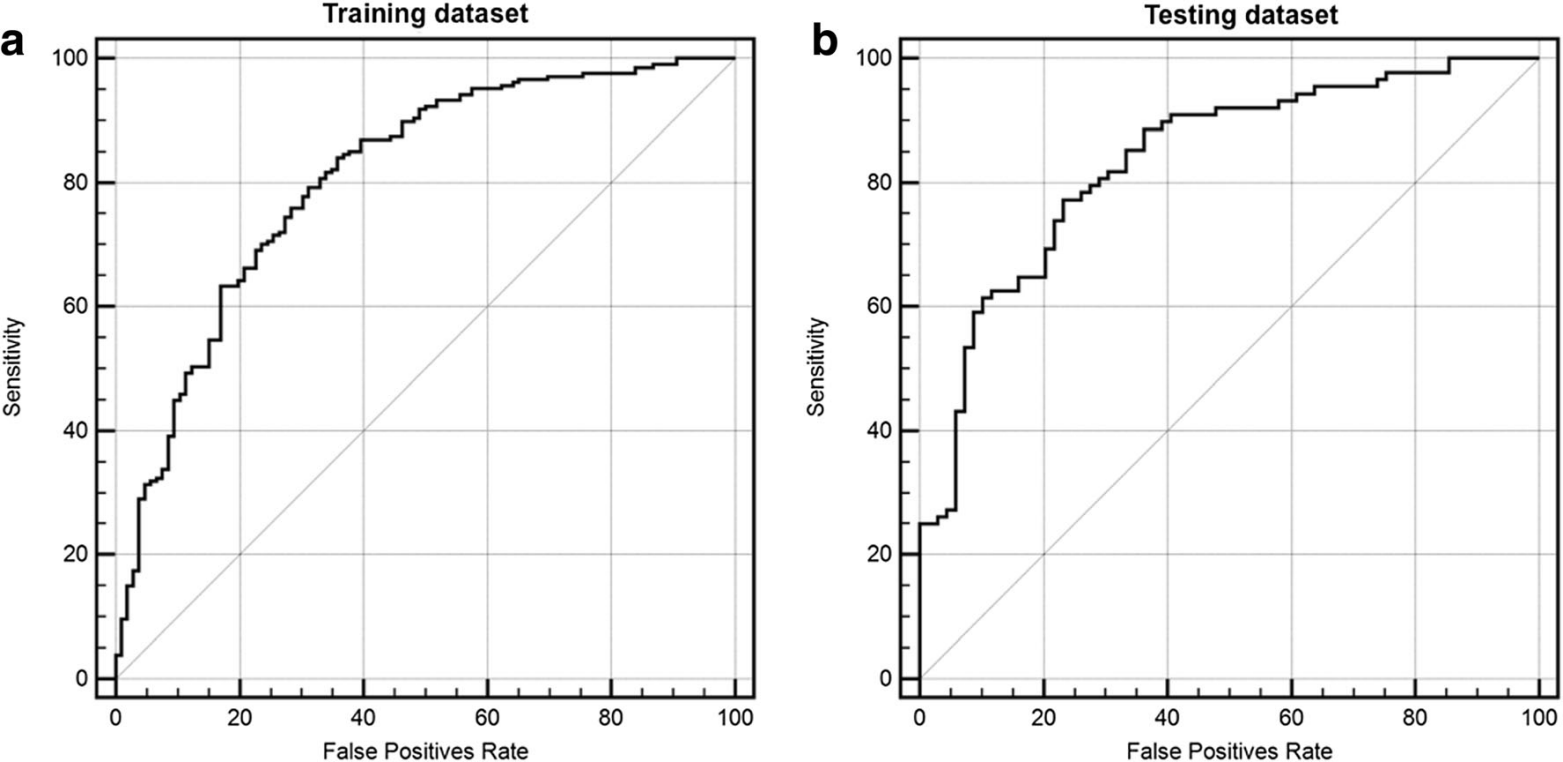
Receiver operating characteristics (ROC) curves for the training (**a**) and testing (**b**) datasets. Detailed results are given in the Results section and Table 2

In 329 patients (mean age 55.1 years, range 18–85 years) included, a total of 470 lesions were histologically verified (Table 1, Fig. 4). Of those, 295 (62.8%) were found to be malignant and 175 (37.2%) benign with a lesion size ranging from 5 to 91 mm. The median lesion size was 16 mm with an interquartile range of 13 mm.

**Table 1:** Histopathological lesion characteristics

			% total	% subgroup
Malignant		295	62.8%	
	Typing			
	IDC	229		76.6%
	ILC	26		8.8%
	DCIS	22		7.5%
	Other	18		6.1%
	Immunohistochemical characteristics			
	HR+, her2neu−	137		46.4%
	HR+, her2neu+	38		12.9%
	HR−, her2neu+	35		11.9%
	HR−, her2neu−	59		20.0%
	Missing/n.a.	26		8.9%
Benign		175	37.2%	
	Fibroadenoma	41		23.4%
	Epithelial proliferations, adenosis	76		43.4%
	Papilloma	33		18.9%
	Phyllodes	2		1.1%
	Inflammation	12		6.9%
	Fibrosis, non-proliferative changes	11		6.3%

*IDC* invasive ductal cancer no specific type (NST), *ILC* invasive lobular cancer, *DCIS* ductal carcinoma in situ, other: invasive mucinous, invasive papillary cancer; malignant phyllodes, metastases; *HR* hormonal receptor; +, positive; −, negative

By means of random allocation, approximately 70% of the lesions were used as training and 30% as testing dataset. Finally, 313 lesions (66.6%, 207 malignant) were assigned as training and 157 (33.7%, 88 malignant) as testing cases.

### Principal component analysis of the extracted features

Eighty-six MRI features were extracted from semi-automatic image analysis. PCA of these features separated 5 main components within the dataset. The component matrix revealed that the main variables influencing component 1 were related to volumetric *k*_trans_ distribution while component 2 was mainly influenced by volumetric *v*_e_ distribution. Component 3 was mainly influenced by the signal intensity changes over time of the maximum wash-out curve and wash-in to wash-out curve and component 4 mainly by the signal intensity changes of the maximum wash-in curve. Finally, component 5 showed major relationships with the lesion volume average signal intensity changes over time (mean curve) and the relative distribution of plateau and persistent curve type voxels (see table, supplemental digital content 2, giving details on component composition).

### Diagnostic performance of the A.I. classifier

The trained multilayer perception MLP 5:3:2 A.I. classifier yielded a highly significant (*p* < .001) AUC of 80.6% (95% CI: 75.8–84.8%). On the testing dataset, the A.I. classifier achieved a highly significant (*p* < .001) AUC of 83.5% (95% CI: 76.8–89.0%). Single predictor importance and A.I. classifier architecture is given in figures supplemental digital content 3 and supplemental digital content 4.

### Potential of the A.I. classifier to avoid unnecessary biopsies

Training set C_1_ was identified at a predicted pseudo-probability of > 0.1741, yielding a sensitivity of 100% and a specificity of 9.4%. C_2_ conditions were fulfilled at a predicted pseudo-probability > 0.2564, achieving a sensitivity of 95.2% and a specificity of 42.5%. At C_1_, 10 of 106 (9.4%) unnecessary biopsies yielding benign results were rated true negative by the ANN classifier, with 0 false negative findings. At C_2_, the number of benign lesions correctly identified as benign was 45/106 (42.5%), yielding 10/207 (4.8%) false negative findings. The majority (8/10) of the false negative lesions were either non-invasive cancers (DCIS, *n* = 6) or low-risk invasive cancers (luminal A type, i.e., ER-/PR-positive, Her2-negative, and low proliferation index Ki-67; *n* = 2). The remaining two false negative lesions were moderately differentiated/intermediate grade (G2) her2-positive invasive lobular cancers.

In the testing sample, evaluating the performance of the predefined A.I. classifier cutoff C_1_ (> 0.1741) led to a sensitivity of 100% and a specificity of 14.5%. Applying C_2_ (> 0.2564) resulted in a sensitivity of 95.5% and a specificity of 36.2%. Ten of 69 (14.5%, C_1_) and 25 of 69 (36.2%, C_2_) of the benign lesions were correctly identified while yielding 0 (C_1_) and four of 88 (4.5%, C_2_) false negative cancers. This resulted in a PPV of 60.0% (C_1_) and 65.6% (C_2_) and a NPV of 100% (C_1_) resp. 86.2% (C_2_) with an accuracy of 62.4% (C_1_) and 69.4% (C_2_). False negative lesions within the testing sample consisted of either non-invasive cancers (DCIS, *n* = 3) or low-risk invasive cancer (NST, well differentiated /low grade, i.e., G1, luminal A type; *n* = 1, Table 2).

**Table 2:** Diagnostic performance of the ANN

	Sensitivity (TP/TP + FN)	95% CI	Specificity (TN/TN + FP)	95% CI	+LR	−LR
Training set (*n* = 313)						
C_1_	100% (207/207)	98.2–100%	9.4% (10/106)	5.2–16.5%	1.1	0
C_2_	95.2% (197/207)	91.3–97.7%	42.5% (45/106)	33.5–52.0%	1.7	0.1
Test set (*n* = 157)						
C_1_	100% (88/88)	95.9–100%	14.5% (10/69)	7.2–25%	1.2	0
C_2_	95.5% (84/88)	88.8–98.7%	36.2% (25/69)	25.0–48.7%	1.5	0.1

*ANN* artificial neural network, *TP* true positive, *TN* true negative, *FP* false positive, *FN* false negative, +/−; *LR* likelihood ratio, C_1_, 100% sensitivity cutoff; C_2_, > 95% sensitivity cutoff

## Discussion

We demonstrate that the investigated temporally and spatially resolved (4D) radiomics approach on DCE images can distinguish benign from malignant enhancing breast lesions. Using a high-sensitivity cutoff for malignancy could potentially have avoided 15% (C_1_) of the biopsies of breast lesions with final benign outcomes without false negatives. The rate of avoidable biopsies could have been increased up to 36.2% (C_2_) at the cost of 3 missed non-invasive DCIS and one missed luminal A type IDC.

In a variety of indications, bMRI is increasingly recognized as a powerful diagnostic tool [1, 2, 33]. Recent years have brought several publications unambiguously demonstrating the added value of bMRI in intermediate-risk screening [3, 4, 34]. These studies pave the ground for tailored screening approaches where bMRI could be applied in women with mammographically extremely dense breasts. One of the major issues when using bMRI as an additional diagnostic tool is the workup of lesions only visible on MRI [2, 33, 35]. While some of these lesions can be visualized by targeted ultrasound examinations, additional second-look ultrasound examinations require substantial personnel, and, though less expensive than MRI-guided biopsies, money. MRI-guided biopsies are effective for diagnosing breast cancer but invasive and time consuming [2, 35]. In addition, a survey by the European Society of Breast Imaging (EUSOBI) pointed out a shortage regarding MRI-guided invasive procedures in Europe [2]. Therefore, methods for avoiding false positive MR BI-RADS category assignments are warranted. Previous research efforts used either further MRI techniques [11–13] or dedicated clinical decision rules based on morphologic and kinetic BI-RADS criteria [14]. While the results of these approaches were encouraging, additional measurements increase magnet time and clinical decision rules require human feature interpretation. Even though clinical decision rules may reduce difficulties in image interpretation, differences due to different experience levels [15] and inter-reader variation remain [16].

Therefore, recent years have seen the rise of quantitative multi-dimensional analysis of imaging data which are considered to reflect underlying phenotypes of neoplastic disease, now referred to as radiomics [36]. There is a growing number of publications on this topic, using variable software systems, data analysis, and classification techniques with different focus and endpoints, making comparison of performance and outcome challenging [37]. Technical issues regarding study comparability include image analysis, preprocessing, normalization, feature reduction, and neural network structure [37–40]. For clinically applicable study results, an endpoint relevant for clinical decision-making should be defined. In clinical management of breast lesions, unnecessary biopsies remain a major clinical issue. To estimate the value of additional tests including radiomic classifiers, high-sensitivity cutoffs in biopsied patient populations may estimate the rate of potentially avoidable biopsies [12, 14, 16, 19, 20, 24]. Using automatic analysis of classical kinetic and pharmacokinetic parameters to build a volumetric 4D radiomic ANN classifier, we found about 15% (C_1_) respectively 36% (C_2_) potentially avoidable biopsies in a setting of MRI-suspicious breast lesions with histological verification. The diagnostic accuracy reported therefore equals the possible improvement of lesion characterization by the established ANN over initial human interpretation (who assigned the initial BI-RADS categories and biopsy recommendations) in the investigated setting. Truhn et al [41] reported on a radiomic and deep learning study to distinguish benign and malignant lesions in bMRI based on T2-weighted and dynamic contrast-enhanced image-derived features. Though their results were encouraging, diagnostic performance estimates were below human readers and the impact of clinical decision-making (i.e., to perform or not perform a biopsy) was not investigated. Advantages of our approach include the following: commercially available software with transparent underlying algorithms and the inclusion of DCE data reflecting physiological information as compared to agnostic criteria without underlying physiological background. Further, we chose a defined and clinically relevant setting and endpoint (avoidable biopsies), a sufficiently large database and a split sample validation. Recently, Verburg et al [5], in a screening setting on women with extremely dense breasts including 85% of benign lesions, found 41.5% respective 26.2% of avoidable biopsies in recalled patients via a radiomic model based on 46 imaging and 3 clinical parameters using a multiparametric or abbreviated MRI protocol. Another study by Illan et al [42] focused on the clinically challenging non-mass lesions in bMRI and provided automatic segmentation, aiding visual analysis of contrast enhancement kinetics for inexperienced and expert readers. Next to facilitating lesion characterization, a radiomics method incorporating prior knowledge on physiological enhancement characteristics has been shown useful for predicting survival in patients with primary breast cancer, based on automatically extracted contrast enhancement kinetics and volumetric features [43].

Vascular properties can be quantified by DCE measurements including pharmacokinetic mapping. The main components of our model were primarily composed of the volumetric characteristics (histogram parameters) of *k*_trans_ (component 1) and *v*_e_ (component 2), which are known to be closely related to vascular net diameter and permeability (*k*_trans_) and extracellular compartment properties (*v*_e_). Notably, and in line with other investigations on malignant tissue characterization, it was not only the parameters themselves but their spatial distribution characteristics that independently contributed to lesion diagnosis, stressing the value of a volumetric approach [27]. The other three identified main components were mostly dependent on enhancement kinetics such as wash-in and wash-out, matching the BI-RADS criteria for raising suspicion for cancer [44].

Some limitations of the presented study have to be addressed. First, our study was designed retrospectively with an inherent selection bias towards clinically challenging cases, which were referred to biopsy. Consequently, the prevalence of malignant lesions in our study is higher compared to the general population. Moreover, the study was conducted in a high prevalence setting resulting in a database that included a mix of lesions that were visible on conventional images or bMRI. Therefore, the results must be called exploratory at this stage and cannot be directly generalized, e.g., to screening recalls. Nevertheless, this design allows to assess a clinically relevant endpoint: avoidable biopsies in benign lesions. Using only MRI-suspicious lesions that underwent histological confirmation results in a database consisting only of true positive and false positive lesions referring to the initial clinical read by the reporting radiologists. Therefore, diagnostic performance estimates directly translate into improved diagnostic accuracy and allow measuring the rate of potentially avoidable biopsies and their costs in false negative results. We did not perform a dedicated reproducibility analysis of the automated lesion segmentation and feature extraction. Our clinical experience with the software used along with the underlying segmentation algorithm suggests very little variation, which might only be possible in very noisy data or very large and ill-defined enhancements. The approach of using a single vendor system on single vendor image data might be considered a limitation. However, the DCE-derived volumetric parameters used for this study did not use higher dimensional texture features that may be prone to vendor-specific bias. While our results that are based on MR images acquired according to international recommendations are encouraging, we can envision an even higher diagnostic potential using MRI techniques achieving higher temporal and spatial resolution. Finally, our exploratory results, though proven robust upon split sample validation, require independent, preferably prospective testing to demonstrate their clinical applicability. In addition, future research may also include a number of other established parameters, such as shape and textural features as well as T2-weighted features [8, 41].

In conclusion, the investigated temporally and spatially resolved (4D) radiomics approach revealed a high diagnostic ability to distinguish between benign and malignant lesions without requiring subjective reader interpretation. Applying the proposed ANN, a relevant number of unnecessary biopsies on benign lesions could have been averted automatically, facilitating the workflow for radiologists and reducing the burden for patients.

## Supplementary Information

ESM 1 (DOCX 462 kb)

## Abbreviations

A.I.: Artificial intelligence
ANN: Artificial neural network
AUC: Area under the curve
BI-RADS: Breast Imaging Reporting and Data System
bMRI: Breast MRI
CLAIM: Checklist for Artificial Intelligence in Medical Imaging
CM: Contrast medium
DCE: Dynamic contrast enhanced
DCIS: Ductal carcinoma in situ
DICOM: Digital Imaging and Communications in Medicine
EES: Extravascular extracellular space
EUSOBI: European Society of Breast Imaging
FDA: US Food and Drug Administration
Gd-DTPA: Gadoteric acid
NST: “No specific type” (former invasive ductal carcinoma or not otherwise specified (NOS))
PACS: Picture Archiving and Communication System
PCA: Principal component analysis
ROC: Receiver operating characteristic
SI: Signal intensity

## Declarations

### Guarantor

The scientific guarantor of this publication is Assoc. Prof. Priv.-Doz. Dr. Pascal A.T. Baltzer.

### Statistics and biometry

Dr. Michael Weber kindly provided statistical advice for this manuscript. One of the authors has significant statistical expertise.

### Methodology

• retrospective

• performed at one institution
