# Supplementary material for: An A.I. classifier derived from 4D radiomics of dynamic contrast-enhanced breast MRI data: potential to avoid unnecessary breast biopsies
Source: Eur Radiol. 2021 Mar 20;31(8):5866–76. doi: 10.1007/s00330-021-07787-z (PMC8270804; doi:10.1007/s00330-021-07787-z)

**Supplemental digital content**

Supplemental digital content 1.docx

Supplemental digital content 1: combination of curve types by initial enhancement (i: slow, ii: medium, iii: fast) and curve type (I: persistent, II: plateau, III: wash-out). 9 different curve types result in voxels passing the 33% initial enhancement threshold.

Supplemental material 1


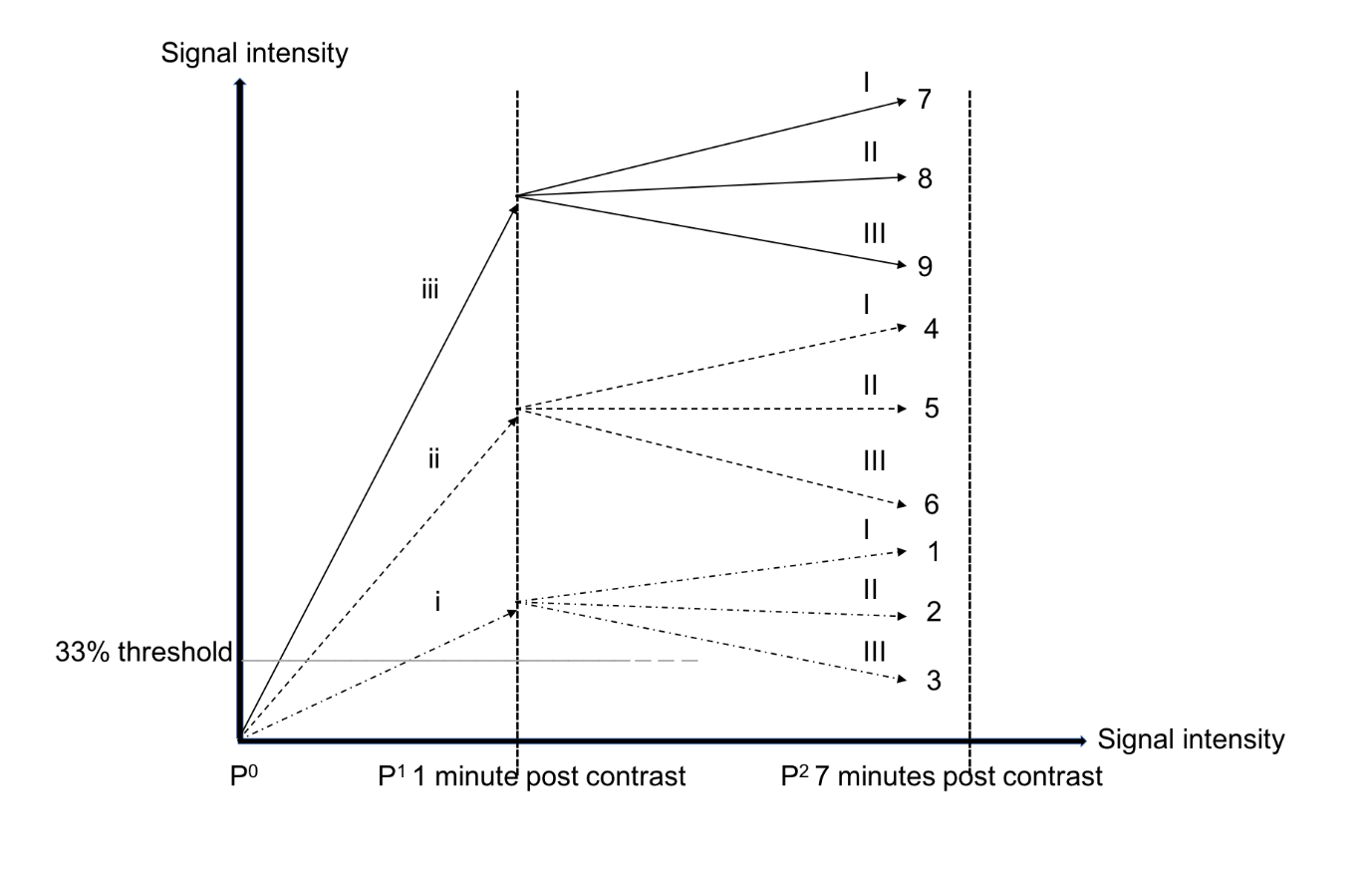


Supplemental scheme 1: combination of curve types by initial enhancement (i: slow, ii: medium, iii: fast) and curve type (I: persistent, II: pleateau, III: wash-out). 9 different curve types result in voxels passing the 33% initial enhancement threshold.

Supplemental digital content 2.docx

Supplemental digital content 2: component matrix showing the individual features and their degrees of contributing to each of the five main components. The 10 most important variables with the highest R^2^ are highlighted.

Supplemental material 2

| **Component Matrix**  (10 most important variables with highest R^2^ **highlighted**) |  |  |  |  |  |
| --- | --- | --- | --- | --- | --- |
|  |  |  |  |  |  |
| **Component** | **1** | **2** | **3** | **4** | **5** |
| relative volume type 1 | -0.687 | 0.451 | -0.121 | -0.1 | **0.462** |
| relative volume type 2 | -0.239 | -0.234 | 0.343 | 0.28 | **-0.596** |
| relative volume type 3 | 0.396 | -0.687 | -0.012 | -0.081 | -0.181 |
| relative volume type 4 | 0.252 | 0.595 | -0.094 | -0.108 | -0.001 |
| relative volume type 5 | 0.514 | 0.417 | -0.018 | 0.111 | -0.317 |
| relative volume type 6 | 0.765 | -0.149 | -0.036 | 0.05 | -0.074 |
| relative volume type 7 | 0.208 | 0.362 | -0.025 | -0.14 | -0.066 |
| relative volume type 8 | 0.422 | 0.374 | 0.011 | 0.03 | -0.094 |
| relative volume type 9 | 0.672 | 0.012 | 0.002 | 0.062 | 0.207 |
| Mean SI precontrast | -0.38 | -0.083 | 0.281 | 0.002 | -0.019 |
| SI precontrast most suspect area | -0.248 | -0.038 | 0.047 | 0.124 | -0.196 |
| Postcontrast SI mean curve 1 | 0.839 | -0.108 | -0.259 | 0.134 | **-0.302** |
| Postcontrast SI mean curve 2 | 0.798 | 0.145 | -0.3 | 0.209 | **-0.301** |
| Postcontrast SI mean curve 3 | 0.749 | 0.278 | -0.321 | 0.208 | **-0.291** |
| Postcontrast SI mean curve 4 | 0.687 | 0.379 | -0.349 | 0.216 | **-0.278** |
| Postcontrast SI mean curve 5 | 0.649 | 0.425 | -0.358 | 0.226 | **-0.273** |
| Postcontrast SI mean curve 6 | 0.605 | 0.475 | -0.373 | 0.225 | **-0.264** |
| Postcontrast SI mean curve 7 | 0.564 | **0.528** | -0.378 | 0.21 | -0.241 |
| Postcontrast SI maximum Wash-in curve 1 | 0.692 | 0.099 | 0.48 | **-0.213** | 0.009 |
| Postcontrast SI maximum Wash-in curve 2 | 0.661 | 0.263 | 0.508 | **-0.373** | -0.009 |
| Postcontrast SI maximum Wash-in curve 3 | 0.584 | 0.305 | 0.497 | **-0.491** | -0.048 |
| Postcontrast SI maximum Wash-in curve 4 | 0.517 | 0.329 | 0.503 | **-0.546** | -0.072 |
| Postcontrast SI maximum Wash-in curve 5 | 0.451 | 0.35 | 0.497 | **-0.583** | -0.113 |
| Postcontrast SI maximum Wash-in curve 6 | 0.421 | 0.36 | 0.47 | **-0.611** | -0.128 |
| Postcontrast SI maximum Wash-in curve 7 | 0.39 | 0.379 | 0.47 | **-0.624** | -0.131 |
| Postcontrast SI maximum Wash-out curve 1 | 0.685 | -0.117 | **0.357** | 0.432 | 0.147 |
| Postcontrast SI maximum Wash-out curve 2 | 0.682 | 0.008 | **0.364** | 0.475 | 0.205 |
| Postcontrast SI maximum Wash-out curve 3 | 0.646 | 0.052 | **0.349** | 0.507 | 0.195 |
| Postcontrast SI maximum Wash-out curve 4 | 0.595 | 0.064 | **0.364** | 0.547 | 0.194 |
| Postcontrast SI maximum Wash-out curve 5 | 0.541 | 0.06 | **0.328** | 0.593 | 0.183 |
| Postcontrast SI maximum Wash-out curve 6 | 0.48 | 0.052 | **0.331** | 0.623 | 0.189 |
| Postcontrast SI maximum Wash-out curve 7 | 0.386 | 0.104 | **0.359** | 0.645 | 0.169 |
| Postcontrast SI maximum Wash-in/Wash-out curve 1 | 0.747 | -0.094 | 0.491 | 0.009 | 0.072 |
| Postcontrast SI maximum Wash-in/Wash-out curve 2 | 0.758 | 0.031 | **0.522** | 0.02 | 0.102 |
| Postcontrast SI maximum Wash-in/Wash-out curve 3 | 0.742 | 0.049 | **0.531** | **0.012** | 0.092 |
| Postcontrast SI maximum Wash-in/Wash-out curve 4 | 0.7 | 0.072 | 0.549 | **0.016** | 0.085 |
| Postcontrast SI maximum Wash-in/Wash-out curve 5 | 0.671 | 0.08 | 0.563 | **0.039** | 0.052 |
| Postcontrast SI maximum Wash-in/Wash-out curve 6 | 0.655 | 0.057 | **0.548** | 0.065 | 0.049 |
| Postcontrast SI maximum Wash-in/Wash-out curve 7 | 0.595 | 0.097 | 0.566 | 0.039 | 0.039 |
| ktrans percentile 10 | 0.652 | -0.442 | -0.279 | -0.207 | 0.092 |
| ktrans percentile 20 | 0.691 | -0.441 | -0.287 | -0.206 | 0.117 |
| ktrans percentile 25 | **0.706** | -0.449 | -0.287 | -0.202 | 0.119 |
| ktrans percentile 30 | **0.714** | -0.448 | -0.284 | -0.203 | 0.126 |
| ktrans percentile 40 | **0.711** | -0.462 | -0.287 | -0.21 | 0.156 |
| ktrans percentile 50 | **0.735** | -0.472 | -0.277 | -0.202 | 0.166 |
| ktrans percentile 60 | **0.738** | -0.498 | -0.263 | -0.195 | 0.184 |
| ktrans percentile 70 | **0.753** | -0.491 | -0.23 | -0.178 | 0.189 |
| ktrans percentile 75 | **0.747** | -0.492 | -0.215 | -0.177 | 0.188 |
| ktrans percentile 80 | **0.744** | -0.479 | -0.198 | -0.168 | 0.176 |
| ktrans percentile 90 | **0.449** | -0.438 | -0.185 | -0.14 | 0.18 |
| ve percentile 10 | 0.393 | 0.601 | -0.364 | 0.022 | 0.133 |
| ve percentile 20 | 0.525 | **0.636** | -0.307 | 0.01 | 0.125 |
| ve percentile 25 | 0.552 | **0.649** | -0.306 | 0.001 | 0.118 |
| ve percentile 30 | 0.593 | **0.659** | -0.291 | 0.003 | 0.104 |
| ve percentile 40 | 0.603 | **0.686** | -0.268 | -0.004 | 0.096 |
| ve percentile 50 | 0.617 | **0.696** | -0.241 | -0.012 | 0.097 |
| ve percentile 60 | 0.634 | **0.698** | -0.192 | -0.005 | 0.092 |
| ve percentile 70 | 0.613 | **0.703** | -0.157 | -0.013 | 0.104 |
| ve percentile 75 | 0.598 | **0.695** | -0.134 | -0.037 | 0.113 |
| ve percentile 80 | 0.589 | **0.697** | -0.11 | -0.054 | 0.113 |
| ve percentile 90 | 0.519 | 0.666 | -0.05 | -0.084 | 0.14 |
| iAUC percentile 10 | 0.786 | -0.256 | -0.257 | -0.164 | -0.047 |
| iAUC percentile 20 | 0.873 | -0.198 | -0.22 | -0.124 | -0.094 |
| iAUC percentile 25 | 0.893 | -0.183 | -0.218 | -0.106 | -0.096 |
| iAUC percentile 30 | 0.905 | -0.157 | -0.206 | -0.094 | -0.108 |
| iAUC percentile 40 | 0.925 | -0.134 | -0.183 | -0.067 | -0.12 |
| iAUC percentile 50 | 0.937 | -0.11 | -0.156 | -0.022 | -0.124 |
| iAUC percentile 60 | 0.943 | -0.09 | -0.128 | 0.008 | -0.123 |
| iAUC percentile 70 | 0.947 | -0.082 | -0.085 | 0.041 | -0.11 |
| iAUC percentile 75 | 0.943 | -0.063 | -0.058 | 0.069 | -0.107 |
| iAUC percentile 80 | 0.937 | -0.051 | -0.035 | 0.086 | -0.104 |
| iAUC percentile 90 | 0.927 | -0.026 | 0.031 | 0.107 | -0.08 |
| relative Wash-out curve type volume | 0.695 | -0.587 | -0.022 | -0.035 | -0.132 |
| relative Plateau curve type volume | 0.034 | -0.005 | 0.319 | 0.309 | **-0.692** |
| relative Persistent curve type volume | -0.646 | 0.531 | -0.132 | -0.116 | **0.457** |
| Wash-out rate maximum Wash-in/Wash-out curve time point 1 | 0.588 | -0.338 | 0.116 | -0.041 | 0.084 |
| Wash-out rate maximum Wash-in/Wash-out curve time point 2 | 0.761 | -0.086 | 0.272 | -0.016 | 0.169 |
| Wash-out rate maximum Wash-in curve time point 1 | 0.082 | -0.415 | -0.206 | 0.639 | 0.181 |
| Wash-out rate maximum Wash-in curve time point 2 | 0.243 | -0.315 | -0.126 | 0.605 | 0.228 |
| Wash-out rate maximum Wash-out curve time point 1 | 0.641 | -0.292 | 0.164 | -0.03 | 0.045 |
| Wash-out rate maximum Wash-out curve time point 2 | 0.731 | -0.105 | 0.215 | 0.077 | 0.158 |
| Wash-out rate mean curve time point 1 | 0.341 | -0.848 | 0.167 | -0.106 | -0.073 |
| Wash-out rate mean curve time point 2 | 0.479 | -0.756 | 0.148 | 0.001 | -0.126 |
| IQR ktrans | **0.724** | -0.48 | -0.19 | -0.164 | 0.195 |
| IQR ve | 0.469 | 0.541 | 0.019 | -0.053 | 0.08 |
| IQR iAUC | 0.864 | -0.001 | 0.023 | 0.143 | -0.101 |

Supplemental digital content 3.png

Supplemental digital content 3: Diagram depicting the normalized importance of each individual identified main component 1 to 5.


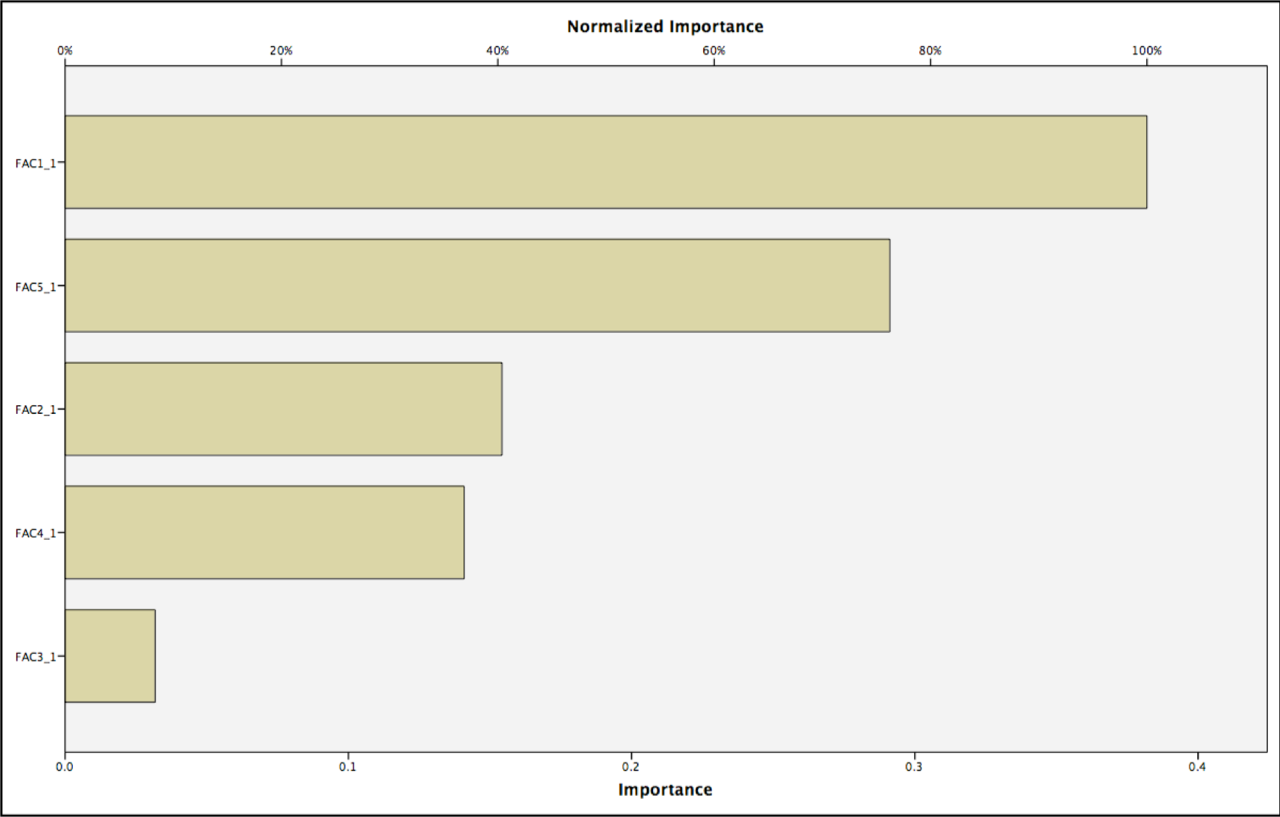


Supplemental digital content 4.png

Supplemental digital content 4: ANN architecture used with one hidden layer based on the prior identified five main components for predicting malignancy of breast lesions.


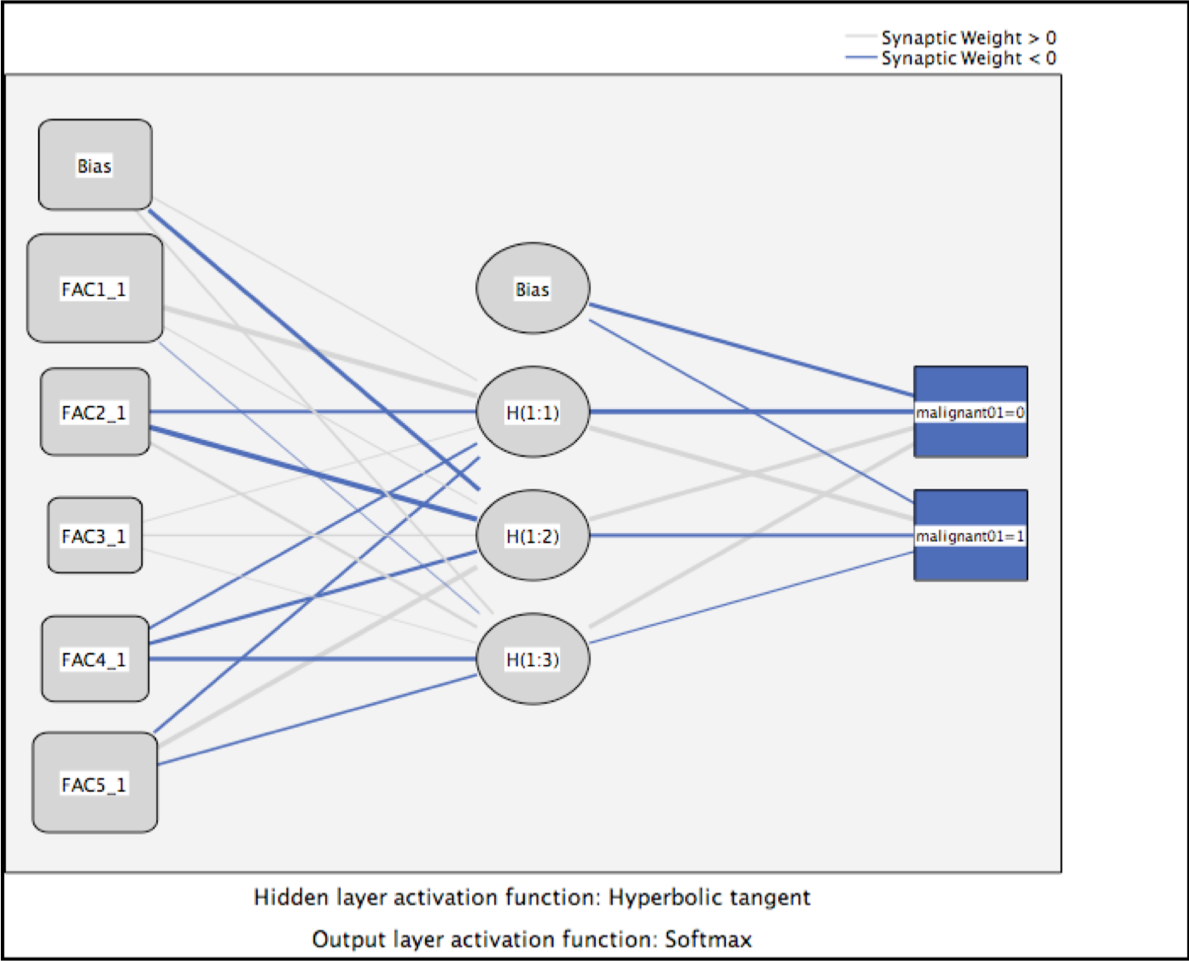

Supplement: Supplementary file 1 — (DOCX 462 kb) [file 330_2021_7787_MOESM1_ESM.docx]
